# Comparison of ERCP Outcomes and Complication Risk between Elderly and Younger Patients: A Large Single-Center Study

**DOI:** 10.3390/jcm13206112

**Published:** 2024-10-14

**Authors:** Yavuz Cagir, Muhammed Bahaddin Durak, Cem Simsek, Ilhami Yuksel

**Affiliations:** 1 Department of Gastroenterology, Ankara Bilkent City Hospital, Ankara 06800, Turkey; yukselilhami@hotmail.com; 2 Department of Gastroenterology, Faculty of Medicine, Hacettepe University, Ankara 06230, Turkey; doctormbd@gmail.com (M.B.D.); cemgsimsek@gmail.com (C.S.); 3 Department of Gastroenterology, Faculty of Medicine, Ankara Yildirim Beyazit University, Ankara 06800, Turkey

**Keywords:** endoscopic retrograde cholangiopancreatography, ERCP procedure-related complications, elderly, common bile duct, periampullary diverticulum

## Abstract

**Objectives:** The current study compared potential risks, complications, and the impact on clinical outcomes among elderly and younger patients undergoing endoscopic retrograde cholangiopancreatography (ERCP). **Methods:** Procedure-related complications, risk factors, and clinical outcomes following complications in elderly patients (aged ≥75 years) and younger who underwent biliary ERCP were evaluated. **Results:** Median age of 63 (48–74) of 1164 patients who underwent biliary ERCP for the first time, and 266 (22.8%) were elderly. Comorbidities were statistically significant (81 [30.5%] versus 78 [8.7%], *p* < 0.001), and periampullary diverticulum (PAD) was detected more commonly in the elderly group (79 [29.7%] vs. 103 [11.5%], *p* < 0.001). There was no statistical difference in cannulation technique, cannulation time, and cannulation success in both groups, while the total ERCP procedure time was higher in the elderly group (22 [16–29] vs. 20 [14–29], *p* = 0.030). Regarding the procedure-related complications, there was no statistically significant difference between the two groups (26 [9.8%] vs. 71 [7.9%], *p* = 0.292). In the case of complications, the length of hospitalization stay was statistically longer in the elderly group. Moreover, the elderly had a longer length of hospitalization, experiencing pancreatitis and a higher probability of developing moderate/severe pancreatitis. In multivariate and univariate analysis, prolonged cannulation time was found to be an independent risk factor in patients ≥75 years of age. **Conclusions:** This study showed that while ERCP-related complication rates in elderly patients are comparable to younger patients, it can be associated with worse outcomes following the complication and prolonged length of hospitalization.

## 1. Introduction

Endoscopic retrograde cholangiopancreatography (ERCP) is generally considered a safe procedure. Nevertheless, despite technological advancements, compliance with safety protocols, and improved endoscopy training programs, ERCP has been associated with a higher prevalence of adverse events compared to most other endoscopic procedures [1,2]. This increased risk may be attributed to ERCP’s evolution into primarily a therapeutic intervention. While the main indication for ERCP procedures remains common bile duct (CBD) stones, it is now widely performed for various conditions such as benign and malignant biliary strictures, chronic pancreatitis, and biliary leaks.

Procedure-related complications are a significant concern for endoscopists. Various studies have reported that the risk of post-ERCP complications ranges from 5 to 12%, even in experienced hands [3,4,5]. These complications are associated with several risk factors, including difficult cannulation, surgically altered anatomy, periampullary diverticulum, sphincter of Oddi dysfunction, and low ERCP case volume centers [6,7,8].

It is reasonable to assume that elderly patients are more likely to require ERCP, given that CBD stones are the most common indication for the procedure, and their incidence increases with age [9]. When considering associated cardiopulmonary disorders, periampullary diverticulum, and other potentially age-related factors, the safety of ERCP becomes even more critical in the elderly population. While the average global life expectancy was 66.2 years in the early 2000s, it is estimated to reach 73.3 years in 2024 and 77 years by 2050 [10]. As the proportion of elderly individuals in the population continues to rise, there is a growing need to develop specific medical strategies tailored to this demographic.

Further research comparing the clinical outcomes and safety of ERCP in elderly patients is essential. The current study not only compares post-ERCP complications between older and younger patients but also aims to identify factors that increase the risk of complications in elderly patients who experience adverse events during or after the procedure.

## 2. Material and Methods

### 2.1. Study Design and Definitions

This retrospective study included ERCP procedures performed by a single experienced endoscopist at Ankara Bilkent City Hospital between January 2021 and March 2024. Patients were divided into two groups: those aged 75 and over, and younger patients. Exclusion criteria encompassed patients under 18 years of age, those with prior sphincterotomy, individuals who underwent percutaneous biliary drainage before ERCP, and those who underwent pancreatic ERCP.

Demographic data, comorbidities, procedure indications, presence of periampullary diverticulum (PAD), cannulation technique, duration, and procedure-related complications were recorded in the electronic medical record system. This study was conducted in accordance with the 1964 Helsinki Declaration, its later amendments, and the institutional scientific committee’s ethical guidelines. This study was approved by the institutional review board (Date of approval: 10 January 2024, number: E2-24-6088).

In geriatric terminology, individuals over 65 are generally categorized as elderly. Recent classifications have further subdivided the elderly population into three distinct groups: the “young-old” (65–74 years), the “middle-old” (75–84 years), and the “oldest-old” (85 years and above). This more nuanced categorization allows for better tailoring of healthcare strategies to the specific needs of each age group [11,12]. Procedure-related complications were defined according to the American Society for Gastrointestinal Endoscopy 2017 guideline [13]. Bleeding was not considered a complication if it was controlled during the procedure, did not cause a drop in hemoglobin level, and did not result in bleeding symptoms. Acute cholangitis diagnosis and severity were determined based on the Tokyo 2018 acute cholangitis guideline [14]. Length of hospitalization was defined as the duration of stay during hospitalization. Mortality was considered as death from any cause during hospitalization, with procedure-related and unrelated deaths distinguished.

### 2.2. ERCP Procedure

All procedures were performed under propofol and meperidine sedoanalgesia, supervised by two nurses and an anesthesiologist. Indomethacin suppositories were administered to all patients before the procedure. When detected, PAD was classified based on the Li-Tanaka classification [15]. In cases of difficult cannulation, alternative methods were employed. The double guide wire approach was used when the guidewire unintentionally entered the pancreatic duct; otherwise, precut sphincterotomy or fistulotomy was attempted based on papilla morphology. Prophylactic pancreatic stents were routinely placed following the double guidewire method. After biliary cannulation, a cholangiogram was obtained. Subsequently, sphincterotomy or a transpapillary balloon dilatation was performed based on the endoscopist’s preference. Biliary stents were inserted as required. Cannulation time, procedure duration, and technical success were documented for each operation.

### 2.3. Statistical Analysis

The Kolmogorov–Smirnov test was used to analyze the normality of continuous variable distribution. Non-normally distributed continuous variables were presented as median (interquartile range) and compared using the Mann–Whitney U test. Categorical variables were expressed as frequency (percentage) and compared via Chi-square or Fisher’s exact test.

Univariate logistic regression analysis, including clinically relevant variables, was performed to determine independent predictors for post-ERCP complications in patients aged ≥75 years. Subsequently, multivariate logistic regression analysis was conducted on variables with a *p*-value < 0.1 in the univariate analysis. Results were presented as odds ratio (OR), 95% confidence interval (CI), and *p*-value. Receiver operating characteristic (ROC) curve analysis was performed to evaluate the ability of continuous independent predictors to forecast post-ERCP complications in patients aged ≥75 years. Results were expressed as the area under the curve (AUC), 95% CI, *p*-value, cut-off value, sensitivity, and specificity. A two-tailed *p*-value < 0.05 was considered statistically significant. Statistical analysis was conducted using IBM SPSS Statistics for Windows, version 25.0 (IBM Corp., Armonk, NY, USA).

## 3. Results

This study included 1164 papilla-naïve patients who underwent ERCP, of whom 664 (57%) were women. The median age was 63 years (IQR: 48–74), with 266 patients (22.8%) aged over 75 years. Among the elderly group, 34 patients were ≥90 years old (nonagenarians). Gender distribution, BMI, and history of past surgeries were comparable between the older and younger cohorts.

Comorbidities were significantly more prevalent in the elderly group (30.5% vs. 8.7%, *p* < 0.001). Regarding procedure indications, malignant biliary strictures were significantly more common in the elderly group (15.4% vs. 9.5%, *p* = 0.006), while biliary leaks were more frequent in the younger group (10.6% vs. 2.6%, *p* < 0.001). PAD was detected more often in the elderly group (29.7% vs. 11.5%, *p* < 0.001).

While there were no significant differences in cannulation technique, cannulation time, and cannulation success between the groups, the total ERCP procedure time was longer in the elderly group (median 22 min [IQR: 16–29] vs. 20 min [IQR: 14–29], *p* = 0.030). When assessing the length of hospital stays, there was no statistically significant difference between the two groups (Table 1). However, among patients who experienced complications, the length of hospitalization was significantly longer in the elderly group (median 10 days [IQR: 8–12.25] vs. 4 days [IQR: 3–5], *p* < 0.001). Furthermore, elderly patients with post ERCP pancreatitis had a longer hospital stay (median 11.5 days [IQR: 9.75–14] vs. 4 days [IQR: 3–5], *p* < 0.001) and a higher probability of developing moderate/severe pancreatitis (50% vs. 6.1%, *p* < 0.001). The overall incidence of procedure-related complications did not differ significantly between the two groups (9.8% vs. 7.9%, *p* = 0.292). No relationship was found between the development of complications and the indication for the procedure in either group. Comorbidities were comparable between patients aged ≥75 and younger who suffered complications (Table 2).

Two elderly patients who developed perforation died due to sepsis despite biliary metal stent placement and extended-spectrum antibiotic treatment. In the younger group, seven patients experienced perforation; one recovered after surgery for duodenal perforation, while six recovered with biliary metal stent placement and antimicrobial treatment. No perforation-related deaths occurred in the younger group. The subgroup analysis of patients aged ≥75 years revealed no significant differences between those who developed complications and those who did not in terms of gender, comorbidities, ERCP indications, presence of PAD, cannulation technique, and cannulation success. However, cannulation time was significantly longer in the group that experienced complications (median 17.5 min [IQR: 5.75–23.25] vs. 9 min [IQR: 5.75–14], *p* = 0.027) (Table 3).

**Figure 1:**
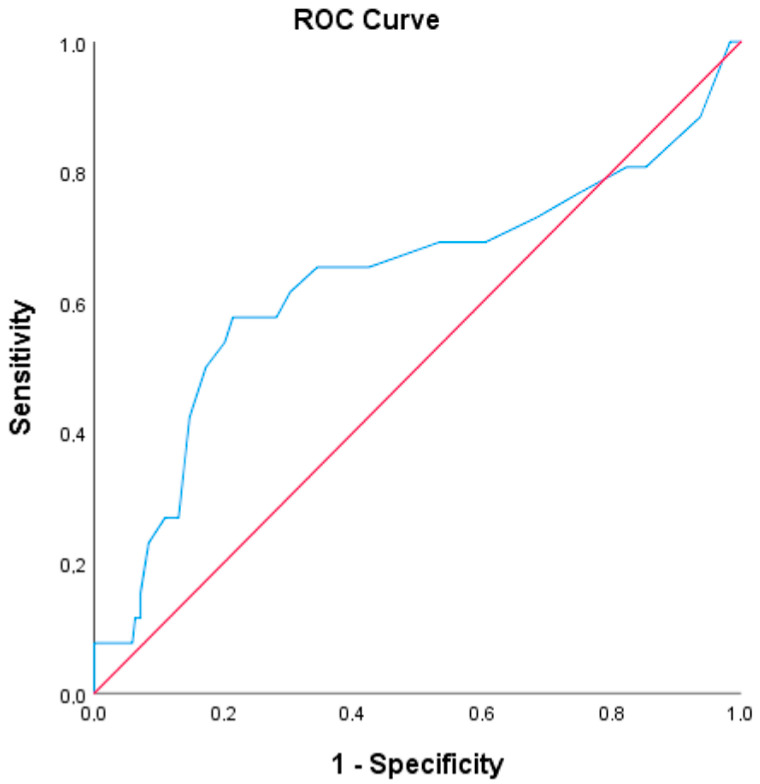
ROC curves of cannulation time in predicting post-ERCP complication occurrence in patients with aged ≥ 75 years.

In determining the risk of procedure-related complications in the ≥75 age group, cannulation time emerged as a significant factor. The OR in the multivariate analysis was 1.060 (95% CI: 1.020–1.101; *p* = 0.003), while the OR in the univariate analysis was 1.061 (95% CI: 1.023–1.101; *p* = 0.002). No significant risk was demonstrated for the remaining parameters (Table 4). The ROC curve analysis revealed that a cut-off cannulation time of ≥15.5 min predicted procedure-related complications with 57.7% sensitivity and 78.6% specificity (Table 5). Figure 1 illustrates the ROC analysis of cannulation time in predicting ERCP-related complications.

## 4. Discussion

As the global population ages, healthcare for the elderly is becoming increasingly significant. Developing new strategies tailored to this demographic is crucial, particularly concerning ERCP procedure-related complications. This study focused on evaluating potential risks, complications, and clinical outcomes in elderly patients undergoing ERCP. Our findings revealed that comorbidities, malignant biliary strictures, and periampullary diverticula were more frequent in the elderly group. While factors such as gender, BMI, past surgery history, cannulation method, and success were comparable between age groups, the total procedure time was longer in the elderly. Although procedure-related complication rates were similar, the elderly group experienced longer hospitalizations when complications occurred, with pancreatitis cases tending to be more severe. Notably, cannulation time emerged as an independent risk factor for complications in the elderly group in both multivariate and univariate analysis.

In the literature, few studies compared ERCP complications between elderly and younger patients. Gardenyes et al. [16] reported no significant difference in complication rates between age groups in their study of 494 patients, with a low 2.6% rate of procedure-associated pancreatitis. Another study focusing on patients over 90 years old found ERCP to be feasible with a low complication rate of 2.5% and mortality rate of 0.7% [17]. Colmonero et al. [10] compared ERCP-related complications in patients over and under 90 years of age in their study of 328 patients, including 28 over 90 years old. They reported no statistical difference in overall adverse events between the groups, despite more frequent sphincterotomy in the over-90 group. Similarly, our study found comparable rates when comparing ages ≥75 years and younger, both post-ERCP pancreatitis (3.8% vs. 3.7%) and overall complication (9.8% vs. 7.9%), respectively. ERCP appears to be generally safe for the elderly population, considering the rates of complications.

The current analysis showed that older patients had longer hospital stays when complications occurred, despite the length of hospital stay being comparable in both groups when comparing the entire population. While overall complication rates were similar across age groups, the older patients tended to have more severe pancreatitis. Notably, while not statistically significant, two elderly patients with perforation died due to sepsis, whereas all seven younger patients with perforation recovered. This higher morbidity and mortality in elderly patients with complications likely reflect weakened immune systems, and overall frailty [16]. In our analysis, comorbidities were not associated with the risk of complications in either group. Since worse outcomes associated with complications in the elderly group in our study were directly related to the procedure, the severity of complications in the elderly may be an indication that these patients are more likely to be affected by more factors than just having additional comorbidities and frailty.

Our study also assessed risk factors predicting complications in the elderly. Prolonged cannulation time (based on cut-off ≥15.5 min) emerged as a significant predictor of increased complication risk. This association is biologically plausible, as extended cannulation can lead to edema, bleeding, and perforation of the papilla [18,19]. Furthermore, alternative cannulation methods were shown to increase procedure-related complications in cases of difficult biliary cannulation [20,21]. The European Society of Gastrointestinal Endoscopy (ESGE) recommends that after more than five contacts with the papilla or a cannulation attempt longer than 5 min, alternative cannulation methods should be used, or the procedure should be postponed/referred to an experienced center [22]. Considering that the development of procedure-related complications is associated with worse outcomes, a more conservative approach may be reasonable in the elderly population. Pavlides et al. [23] performed precut sphincterotomy in 187 patients following prolonged biliary cannulation. In 42% of patients, biliary cannulation was achieved during the first session; in procedures subsequently postponed for a median of 4 days, the success rate was as high as 82%. In our clinical experience, difficult biliary cannulation was experienced in a total of 362 (31%) patients, 68 (19%) of whom were elderly patients. The procedure was performed successfully by using alternative methods in 358 (98.9%) patients with difficult biliary cannulation. Alternative cannulation method details in these patients were as follows: Needle-knife fistulotomy 13.3%, double guide wire (DGW) 7.6%, DGW + transpancreatic biliary sphincterotomy (TPBS) 4.6%, precut sphincterotomy 4.2% and TPBS 1%. There was no difference between the elderly and younger groups regarding difficult biliary cannulation rate (25.6% vs. 32.3%, respectively). Few patients (3.7%) were postponed to the second ERCP session. Our analysis showed that the successful difficult biliary cannulation rate in the first procedure was higher than in previous studies [20,21,22,23]. The possible explanation could be that all procedures were performed by a single experienced endoscopist and in a high-volume tertiary referral center.

The increased frequency of PAD with age is well established [15]. While some studies associated PAD with difficult biliary cannulation [24,25,26], others suggested it may facilitate the procedure by straightening the common bile duct axis [27,28]. In our study, despite a higher prevalence of PAD in the elderly group, cannulation success rates were comparable between elderly and younger groups (99.2% vs. 99.8%, respectively).

In the present study, the higher rate of biliary leaks in the younger group, contrasting with more malignant biliary strictures in the elderly, can be attributed to several factors. Advanced age is a known risk factor for cholangiocarcinoma, gallbladder tumors, and periampullary tumors, explaining the higher incidence of malignant biliary strictures in older patients [29]. Conversely, older individuals undergo cholecystectomy less frequently due to comorbidities, potentially accounting for the lower rate of biliary leakage procedures in this group [30,31]. The longer overall ERCP time in the elderly group, despite similar cannulation durations, may be explained by the higher prevalence of malignant biliary strictures, which often require more extensive procedures post-cannulation [32,33]. Additionally, although not evaluated in this study, potentially larger and more numerous CBD stones in older patients could have contributed to extended procedure times [34].

Our study has several limitations. Its retrospective design and setting in a tertiary ERCP center may have introduced selection bias towards more complex cases. Additionally, the lack of documentation on CBD stone size and number in procedures performed for this indication could have biased the procedure time analysis. However, this study’s strengths lie in its large cohort of elderly patients and the detailed subgroup analysis of those who developed procedure-related complications.

## 5. Conclusions

This study demonstrated a higher prevalence of PAD and longer total ERCP procedure times in the elderly group. Despite these differences, ERCP-related complication rates were similar between elderly and younger patients. However, when complications did occur, elderly patients experienced longer hospitalizations and an increased risk of developing moderate to severe pancreatitis. While ERCP appears to be generally safe for the elderly population, our findings emphasize the importance of careful monitoring and management during hospitalization, particularly when complications arise. Healthcare providers should be prepared for potentially more complex and prolonged care in elderly patients undergoing ERCP, especially in the event of complications.

## Figures and Tables

**Table 1:** Demographic characteristics, clinical variables, and ERCP procedure details in patients aged ≥75 years and in the younger group.

	Total (n = 1164)	Age ≥ 75 Years (n = 266)	Age < 75 Years (n = 898)	*p*-Value
**Gender, female, n (%)**	664 (57)	163 (61.3)	501 (55.8)	0.112
**Age, years**	63 (48–74)	81 (78–86)	58 (44–66)	**<0.001**
**Patients’ BMI, kg/m^2^**	27.15 (25.28–29.65)	27.14 (24.69–30.47)	27.16 (25.39–29.39)	0.654
**Comorbidity, n (%)**	159 (13.7)	81 (30.5)	78 (8.7)	**<0.001**
Cardiovascular Diseases	72 (6.2)	37 (13.9)	35 (3.9)	**<0.001**
Chronic Lung Diseases	60 (5.2)	26 (9.8)	34 (3.8)	**<0.001**
Chronic Kidney Diseases	27 (2.3)	18 (6.8)	9 (1)	**<0.001**
**Past surgery history, n (%)**				
History of cholecystectomy	293 (25.2)	58 (21.8)	235 (26.2)	0.150
Operation Billroth I and II	6 (0.5)	2 (0.8)	4 (0.4)	0.625
**Indications of ERCP procedure, n (%)**				**<0.001**
Choledocholithiasis	850 (73)	206 (77.4)	644 (71.7)	0.064
Malignant biliary strictures	126 (10.8)	41 (15.4)	85 (9.5)	**0.006**
Benign biliary strictures	60 (5.2)	8 (3)	52 (5.8)	0.071
Biliary leak	102 (8.8)	7 (2.6)	95 (10.6)	**<0.001**
Others	26 (2.2)	4 (1.5)	22 (2.4)	0.481
**Periampullary diverticulum, n (%)**	182 (15.6)	79 (29.7)	103 (11.5)	**<0.001**
1	23 (2)	10 (3.8)	13 (1.4)	**0.017**
2A	37 (3.2)	20 (7.5)	17 (1.9)	**<0.001**
2B	64 (5.5)	24 (9)	40 (4.5)	**0.004**
3	33 (2.8)	17 (6.4)	16 (1.8)	**<0.001**
4A	15 (1.3)	5 (1.9)	10 (1.1)	0.331
4B	10 (0.9)	3 (1.1)	7 (0.8)	0.704
**Cannulation techniques, n (%)**				0.162
Standard	802 (68.9)	196 (73.7)	606 (67.5)	0.055
Precut	49 (4.2)	7 (2.6)	42 (4.7)	0.145
Needle-knife fistulotomy	155 (13.3)	29 (10.9)	126 (14)	0.187
Double guide wire (DGW)	88 (7.6)	22 (8.3)	66 (7.3)	0.618
Trans-pancreatic biliary sphincterotomy (TPBS)	12 (1)	1 (0.4)	11 (1.2)	0.316
DGW + TPBS	54 (4.6)	9 (3.4)	45 (5)	0.268
**Cannulation time, min**	9 (5–16)	9 (6–15.75)	9 (4–17)	0.574
**Cannulation success rate, n (%)**	1160 (99.7)	264 (99.2)	896 (99.8)	0.226
**Total ERCP procedure time, min**	21 (15–29)	22 (16–29)	20 (14–29)	**0.030**
**Length of hospital stay, day**	4 (2–6)	4 (1.75–7)	4 (3–5)	0.220

**Table 2:** Subgroup analysis of complications in patients aged ≥75 years and in the younger group.

	Total Complications (n = 97)	Age ≥ 75 Years (n = 26)	Age < 75 Years (n = 71)	*p*-Value
**Comorbidity, n (%)**	6 (6.2)	3 (11.5)	3 (4.2)	0.338
Cardiovascular Diseases	3 (3.1)	1 (3.8)	2 (2.8)	1
Chronic Lung Diseases	1 (1)	-	1 (1.4)	1
Chronic Kidney Diseases	2 (2.1)	2 (7.7)	-	0.069
**Indications of ERCP procedure, n (%)**				0.153
Choledocholithiasis	61 (62.9)	18 (69.2)	43 (60.6)	0.434
Malignant biliary strictures	14 (14.4)	6 (23.1)	8 (11.3)	0.143
Benign biliary strictures	7 (7.2)	-	7 (9.9)	0.184
Biliary leak	10 (10.3)	2 (7.7)	8 (11.3)	1
Others	5 (5.2)	-	5 (7)	0.320
**Post ERCP complications, n (%)**	97 (9.7)	26 (9.8)	71 (7.9)	0.292
Post ERCP Pancreatitis	43 (3.7)	10 (3.8)	33 (3.7)	0.949
Perforation	9 (0.8)	2 (0.8)	7 (0.8)	1
Bleeding	37 (3.2)	12 (4.5)	25 (2.8)	0.158
Cholangitis	3 (0.3)	-	3 (0.3)	1
Others (Cardiopulmonary)	2 (0.2)	-	2 (0.2)	1
Death	3 (0.3)	2 (0.8)	1 (0.1)	0.133
**Length of hospital stay, day**	5 (3–8)	10 (8–12.25)	4 (3–5)	**<0.001**
**Length of hospital stay in patients with pancreatitis, day**	4 (3–9)	11.5 (9.75–14)	4 (3–5)	**<0.001**
**Severity of pancreatitis**				**<0.001**
Mild	33 (76.7)	2 (20)	31 (93.9)	
Moderate/Severe	10 (23.3)	8 (80)	2 (6.1)	

**Table 3:** Demographics, clinical features, and ERCP results of subgroups according to postprocedural complications in patients with aged ≥75 years.

	No Complications (n = 240)	Patients with Complications (n = 26)	*p*-Value
**Gender, female, n (%)**	148 (61.7)	15 (57.7)	0.693
**Age, years**	81 (78–86)	79 (77–85.25)	0.370
**Patients’ BMI, kg/m^2^**	27.14 (24.69–30.47)	26.3 (24.49–28.76)	0.502
**Comorbidity, n (%)**	74(30.8)	7 (26.9)	0.727
Cardiovascular Diseases	36 (15)	4 (15.4)	0.944
Chronic Lung Diseases	22 (15.7)	-	0.088
Chronic Kidney Diseases	16 (6.7)	3 (11.5)	0.191
**Past surgery history, n (%)**			
History of cholecystectomy	50 (20.8)	8 (30.8)	0.244
Operation Billroth I and II	2 (0.8)	-	1
**Indications of ERCP procedure, n (%)**			0.244
Choledocholithiasis	188 (78.3)	18 (69.2)	0.291
Malignant biliary strictures	35 (14.6)	6 (23.1)	0.255
Benign biliary strictures	8 (3.3)	-	1
Biliary leak	5 (2.1)	2 (7.7)	0.142
Others	4 (1.7)	-	1
**Periampullary diverticulum, n (%)**	71 (29.6)	8 (30.8)	0.900
1	9 (3.8)	1 (3.8)	1
2A	17 (7.1)	3 (11.5)	0.426
2B	22 (9.2)	2 (7.7)	1
3	17 (7.1)	-	0.388
4A	3 (1.3)	2 (7.7)	0.077
4B	3 (1.3)	-	1
**Cannulation techniques, n (%)**			0.887
Standard	177 (73.8)	19 (73.1)	
Alternative cannulation methods	61 (25.4)	7 (26.9)	
**Cannulation time, min**	9 (5.75–14)	17.5 (5.75–23.25)	**0.027**
**Cannulation success rate, n (%)**	238 (99.2)	26 (100)	1
**Total ERCP procedure time, min**	22 (16–28)	27 (12–36.25)	0.697

**Table 4:** Univariate and multiple variate logistic regression analysis of independent predictors for post ERCP complications in patients aged ≥75 years.

	Univariate Analysis				Multiple Variate Analysis			
		95% CI				95% CI		
	OR	Lower	Upper	*p*	OR	Lower	Upper	*p*
**Gender, male**	1.180	0.519	2.680	0.693	-	-	-	-
**Age**	0.971	0.899	1.049	0.457	-	-	-	-
**BMI**	0.997	0.904	1.098	0.947	-	-	-	-
**Comorbidity**	0.271	0.079	0.930	**0.038**	0.279	0.080	0.971	**0.045**
**History of cholecystectomy**	1.689	0.694	4.109	0.248	-	-	-	-
**Operation Billroth I and II**	0	0	-	0.999	-	-	-	-
**Indications**					-	-	-	-
Choledocholithiasis	1	-	-	-				
Malignant biliary strictures	1.790	0.664	4.828	0.250				
Benign biliary strictures	0	0	-	0.999				
Biliary leak	4.178	0.756	23.090	0.101				
Others	0	0	-	0.999				
**Periampullary diverticulum**	1.058	0.440	2.545	0.900	-	-	-	-
**Cannulation techniques**					-	-	-	-
Standard	1	-	-	-				
Alternative cannulation methods	1.069	0.429	2.667	0.886				
**Cannulation time**	1.061	1.023	1.101	**0.002**	1.060	1.020	1.101	**0.003**
**Cannulation success**	>100	0	-	0.999	-	-	-	-
**Total ERCP procedure time**	1.012	0.975	1.051	0.526	-	-	-	-

**Table 5:** ROC curve analysis results of cannulation time in predicting post-ERCP complication occurrence in patients with aged ≥75 years.

	AUC	%95 CI		*p* Value	Cut-Off Value	Sensitivity	Specificity
		Lower	Upper				
**Cannulation time**	0.632	0.497	0.767	**0.027**	15.5	0.577	0.786

## Data Availability

The data underlying this article will be shared upon reasonable request to the corresponding author.
